# Circulating tumor DNA as a dynamic biomarker of response to palbociclib and fulvestrant in metastatic breast cancer patients

**DOI:** 10.1186/s13058-021-01411-0

**Published:** 2021-03-06

**Authors:** Lauren Darrigues, Jean-Yves Pierga, Alice Bernard-Tessier, Ivan Bièche, Amanda Bartolini Silveira, Marc Michel, Delphine Loirat, Paul Cottu, Luc Cabel, Coraline Dubot, Romain Geiss, Francesco Ricci, Anne Vincent-Salomon, Charlotte Proudhon, François-Clément Bidard

**Affiliations:** 1 Circulating Tumor Biomarkers Laboratory, Inserm CIC-BT 1428, Institut Curie, PSL Research University, 26 rue d’Ulm, 75005 Paris, France; 2 Department of Surgical Oncology, Institut Curie, Paris, France; 3 Université de Paris, Paris, France; 4 Department of Medical Oncology, Institut Curie, 35 rue Dailly, 92210 Saint Cloud, France; 5 Department of Biopathology, Institut Curie, Paris, France; 6 INSERM U934 CNRS UMR3215, Paris, France; 7 UVSQ Paris-Saclay University, Saint Cloud, France

**Keywords:** Circulating tumor DNA, Breast cancer, Palbociclib-fulvestrant, Treatment follow-up, Precision medicine

## Abstract

**Background:**

Following the PALOMA-3 study results, the combination of palbociclib, a CDK4/6 inhibitor, with fulvestrant, a selective estrogen receptor degrader, has become a standard therapy in women with estrogen receptor-positive (ER+) HER2-negative (HER2−) metastatic breast cancer (MBC). Palbociclib has been shown to increase the progression-free survival (PFS) overall but no predictive biomarker of palbociclib efficacy has been validated so far. We thus evaluated whether early changes of circulating tumor DNA (ctDNA) levels are associated with palbociclib plus fulvestrant efficiency.

**Methods:**

ER+ HER2− MBC patients were included in a prospective observational cohort before treatment initiation. Tumor response was assessed by radiological evaluation (RECIST v1.1) every 3 months. Plasma samples were collected before treatment (baseline), at day 15 (D15), at day 30 (D30), and at disease progression. We searched for somatic mutations from archived tumor tissues by targeted deep sequencing. For patients with somatic mutations identified, circulating tumor DNA (ctDNA) was tracked using digital droplet PCR. Ratios of ctDNA levels ([D15/baseline] and [D30/baseline]) were then correlated with prospectively registered patient characteristics and outcomes.

**Results:**

Twenty-five of the 61 patients enrolled had a somatic mutation testable in plasma (*N*_*PIK3CA*_ = 21, *N*_*TP53*_ = 2, *N*_*AKT1*_ = 2). At baseline, 84% of patients had detectable ctDNA levels but ctDNA levels had no prognostic impact on PFS (*p = 0.10*). Among those patients, ctDNA was still detected in 82% at D15 and 68% at D30. ctDNA clearance observed at day 30 was associated with longer PFS (HR = 7.2, 95% CI = 1.5–32.6, *p* = 0.004). On the contrary, a [D30/baseline] ctDNA ratio > 1 was associated with a shorter PFS (HR = 5.1, 95% CI = 1.4–18.3, *p = 0.02*) and all 5 patients with increased ctDNA levels at D30 showed disease progression after 3 months under palbociclib-fulvestrant. Finally, at the time of radiological tumor progression, ctDNA was detected in all patients tested.

**Conclusion:**

Our study demonstrates that the efficiency of palbociclib and fulvestrant can be monitored by serial analyses of ctDNA before radiological evaluation and that early ctDNA variation is a prognostic factor of PFS.

**Supplementary Information:**

The online version contains supplementary material available at 10.1186/s13058-021-01411-0.

## Introduction

CDK4/6 inhibitors target the proliferative function of cyclin D-associated kinases to induce cell-cycle exit. Beyond the enforcement of cytostatic growth arrest, these drugs may also induce metabolism changes and increase cancer cell immunogenicity [1]. In pre-treated estrogen receptor-positive (ER+), HER2-negative (HER2−) metastatic breast cancer (MBC), pivotal phase 3 trials demonstrated that adding CDK4/6 inhibitors to the hormone-therapy agent fulvestrant led to significant improvement of progression-free survival (PFS) [2–4]. In the PALOMA-3 study, patients treated by combination of palbociclib, the first-in-class CDK4/6 inhibitor, and fulvestrant had a median PFS of 9.5 months, while those treated by placebo and fulvestrant had a median PFS of 4.5 months [2]. In that context, CDK4/6 inhibitors plus fulvestrant became a standard of care for virtually all pre-treated ER+ HER2− MBC patients who did not receive CDK4/6 inhibitor previously. However, some patients may not respond to this therapy. In the PALOMA-3 study, about 21% of patients treated with palbociclib and fulvestrant experienced a PFS shorter than 3 months.

In the absence of available predictive biomarkers, a promising strategy is to monitor treatment-related early changes of blood biomarkers, such as circulating tumor cells [5] or circulating tumor DNA (ctDNA) [6, 7]. ctDNA corresponds to DNA fragments carrying tumor-specific alterations, which is a variable and generally limited fraction of total cell-free circulating DNA (cfcDNA) found in patients’ blood. In early breast cancer, quantitative detection of ctDNA levels has been correlated to patient’s outcome in the context of neoadjuvant therapy, post-neoadjuvant therapy and during follow-up [7–9]. In MBC, ctDNA changes was associated with the survival of patients treated by chemotherapy or targeted therapy [10–12].

To investigate whether ctDNA changes during the first month of palbociclib-fulvestrant is associated with treatment efficacy, we implemented, in April 2016, a prospective cohort of pre-treated ER+ HER2− MBC patients that were about to start this new treatment combination. In 2018, a study by O’Leary et al., detecting PIK3CA ctDNA from plasma collected as part of the PALOMA-3 study, showed that relative change in ctDNA levels after 15 days of treatment predicts PFS on palbociclib and fulvestrant [13]. Here, we further evaluated the early dynamics of ctDNA exploring 15 driver breast cancer genes and assessing further time points: after 30 days of treatment and at the time of progression.

## Methods

### Samples and patients

After written informed consent, patients were included into the prospective, ethically approved, ALCINA study (NCT02866149, cohort #6). Eligibility criteria were as follows: patients aged ≥ 18 years with ER+, HER2− MBC, treated at Institut Curie (Paris and Saint Cloud, France) who progressed under endocrine therapy and for which a treatment with palbociclib and fulvestrant was being initiated. Tumor response to therapy was assessed at least every 3 months and classified, per RECIST v1.1 criteria, as complete response (CR), partial response (PR), stable disease (SD), or progressive disease (PD). For each patient, up to 4 blood samples were collected: before treatment (baseline—bsl), after 15 days (D15), after 30 days (D30), and at time of progression disease (PD) (Supplemental Fig. 1 presents the study workflow).

### Identification of trackable somatic mutations

Archived tumor samples, either from the primary tumor or a metastatic deposit, were retrieved from the Pathology department. Tumor DNA was extracted from macro-dissected tumor tissue and subjected to targeted next generation sequencing (NGS) exploring 15 breast cancer genes (*AKT1*, *ALK*, *BRAF*, *ERBB2*, *EGFR*, *FBXW7*, *HRAS*, *KIT*, *KRAS*, *MAP2K1*, *MAP2K2*, *MET*, *NRAS*, *PDGFRA*, *PIK3CA*). Known activating mutations occurring in these genes were considered as driver genes with a likely clonal distribution, suitable for ctDNA detection.

### Analysis of circulating cell-free and tumor DNA (cfcDNA and ctDNA)

At each time point, 14 mL of blood were drawn in EDTA tubes and processed within 4 h at the Circulating Tumor Biomarkers laboratory. For plasma sample preparation, blood was centrifuged at 820*g* for 10 min to isolate plasma from red blood cells, the supernatant was then transferred to sterile tubes before being centrifuged again at 16,000*g* for 10 min and stored at − 80 °C. cfcDNA was extracted from 4 ml of plasma using the QiaSymphony automated system and the QIAamp Circulating Nucleic Acid kit (Qiagen). Resulting cfcDNA was stored at -20 °C until needed.

For patients with no trackable mutation identified from their tumor tissue, targeted-NGS was performed on their cfcDNA with a panel of 39 cancer-related genes (Supplemental Table 1), developed *in house* and for which library preparation and analysis protocols were reported previously [14, 15].

Droplet digital PCR (ddPCR) analyses were performed on a QX100 ddPCR system (Bio-Rad). PCR was performed in a 20 μL final volume including 10 μL of 2X Supermix for probes without dUTP (Bio-Rad), 1 μL of a 20X mutant primers/probes and wild-type primers/probes mix (18 μM of primers, 5 μM of probes), a variable volume of DNA sample and nuclease-free water up to 20 μL. 1.1 μL of DNA was used for tissue samples and a median of 9 μL for plasma DNA. The PCR reaction was partitioned into a mean of 13,000 droplets per sample using the QX100 Droplet Generator (Bio-Rad) according to the manufacturer’s instructions. Droplets were then transferred to a 96-well PCR plate, placed in a thermocycler, and subjected to the following program: 95 °C for 10 min, 40 cycles of 94 °C for 30 s and hybridization at a specific temperature for each mutation for 60 s, followed by a 10-min incubation at 98 °C. The hybridization temperatures used were as follows: *AKT1* E17K = 55 °C, *TP53* R175H or H179R = 55 °C, *PIK3CA* H1047L = 55 °C, *PIK3CA* H1047R = 60 °C, and *PIK3CA* E545K and E542K = 62 °C. Droplets fluorescence intensity was then analyzed with the laser-equipped QX100 Droplet Reader and the QuantaSoft software v1.4.0.99 from Bio-Rad. The threshold distinguishing positive and negative droplets has been determined manually on the QuantaSoft software by the operator using positive controls on tumor tissue or cell lines for each patient. To ensure uniformity, the threshold obtained was kept for the analysis of all following samples with the same mutation (plasmas and negative controls).

### Statistical analyses

This hypothesis-generating study had no prespecified power. The objectives of the study were to evaluate the relationship between ctDNA detection and variation from baseline (bsl) to D15 and D30 with the tumor response to palbociclib-fulvestrant, as well as the prognostic impact. ctDNA dynamics was evaluated by comparing its variations under treatment using ctDNA ratios defined as ctDNA level at D15 or D30 relative to baseline (bsl). The Wilcoxon test was used to compare cfcDNA and ctDNA levels at the different time points. Progression-free survival (PFS), defined as the time from inclusion in the study to progression disease or death from any cause, was collected prospectively. For the current analysis, disease progression status was determined at the 3-month evaluation dividing patients in 2 groups with progressive disease (PD) versus non-PD patients, respectively. Survival analysis was performed using Kaplan–Meier plots with significance tested using the log-rank test. Statistical analyses were performed with GraphPad Prism (version 8.0) and R (version 3.2.2). All tests were 2-sided.

## Results

### Identification of trackable somatic mutations

**Fig. 1:**
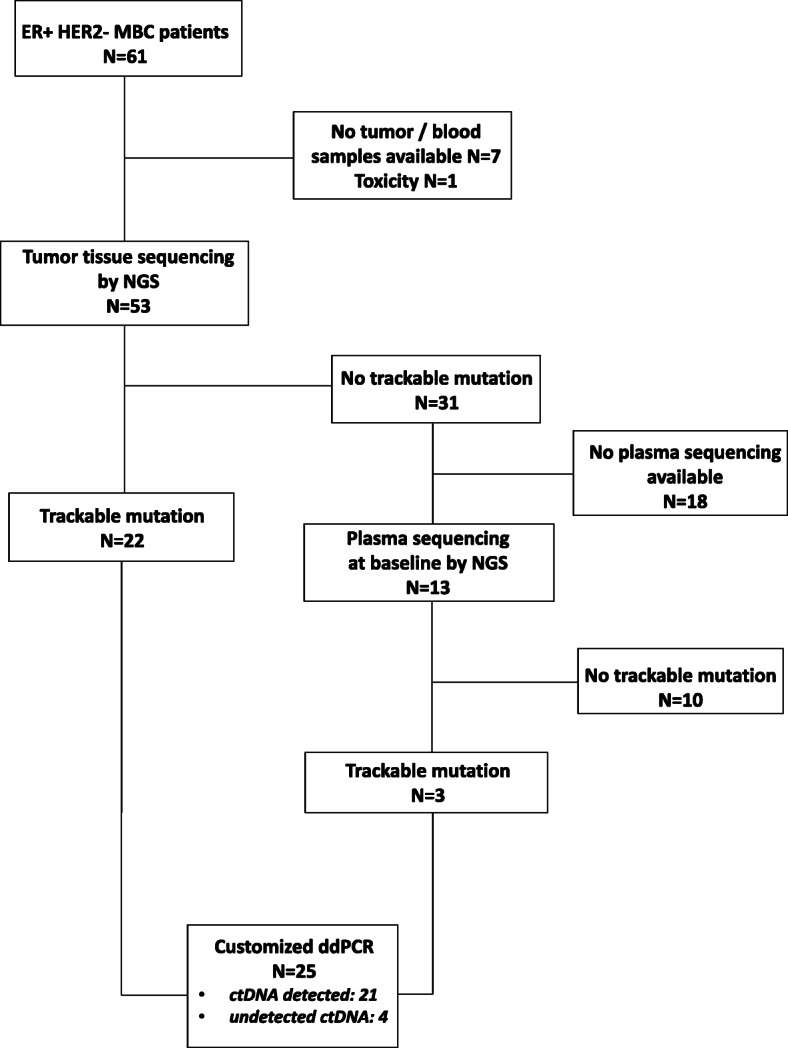
Identification of trackable somatic mutations. Study flow chart. N = number of patients

A total of 61 ER+ HER2− MBC patients were included in the ALCINA (cohort 6) study from June 2016 to March 2018. Patients were pretreated with a median number of 3 lines of treatment (range = 1–8, endocrine therapy or chemotherapy). Median age was 69 (range = 48–80). At the time of analysis (Oct 2018), 53 patients had progressed under treatment and 11 had died. Fifty-three patients had an archived tumor tissue available, either from the primary tumor or biopsies of metastases. Exploring 15 driver breast cancer genes by NGS, we identified 22 patients with one or two somatic mutations that were trackable in cfcDNA by ddPCR. Further targeted NGS, performed on baseline plasma samples, identified 3 additional patients with trackable mutations. In total, 25 patients (41%) were assessed for ctDNA detection from their plasma (Fig. 1, Supplemental Table 2, *PIK3CA N* = 21, *TP53* N = 2, *AKT1* N = 2).

### ctDNA detection at baseline

**Fig. 2:**
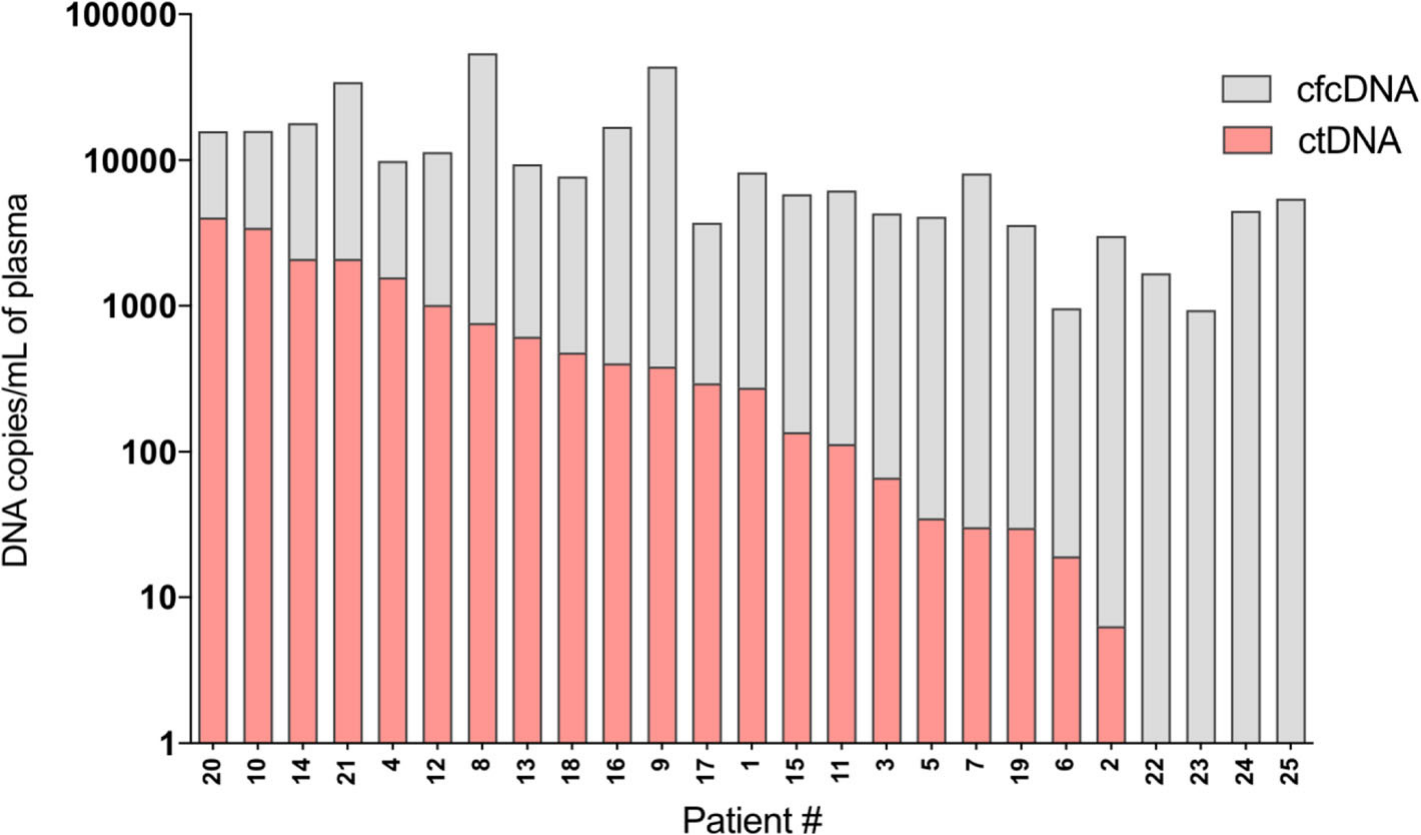
ctDNA detection at baseline. cfcDNA and ctDNA concentrations, prior palbociclib-fulvestrant treatment initiation, are displayed in copies per ml of plasma extracted. Patients are ranked by decreasing ctDNA concentrations. Gray bars represent cfcDNA concentrations; pink bars represent ctDNA concentrations

Using ddPCR assays targeting the mutations identified by NGS, we quantified the level of mutant and wild-type (WT) circulating DNA copies. At baseline, cfcDNA, corresponding to mutant and WT copies, was detected in all 25 patients whereas mutant copies (ctDNA) were detected only in 21 patients (84%) (Fig. 2). In patients with detected ctDNA, the median allelic frequency was 2.5% (range = 0.2–34.6%) (Supplemental Table 2). No correlation was observed between ctDNA and cfcDNA levels at baseline. As previously observed by O’Leary et al. [13], baseline ctDNA levels had no prognostic impact on PFS (Supplemental Fig. 2, > vs ≤ median, HR = 1.75, 95% CI = 0.7–4.4, *p* = 0.1).

### Early ctDNA dynamics during palbociclib-fulvestrant treatment and impact on survival

**Fig. 3:**
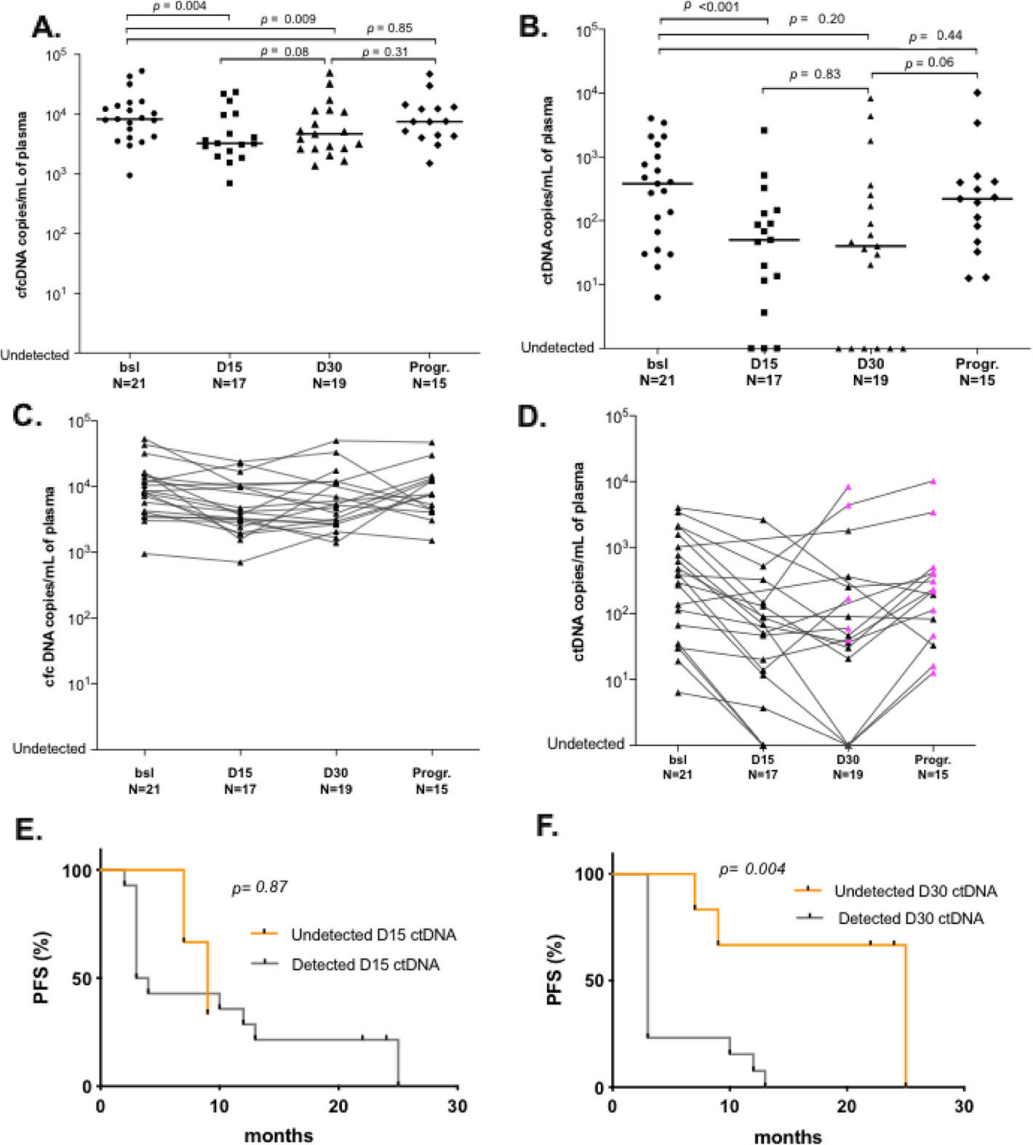
Early ctDNA dynamics during palbociclib-fulvestrant treatment and impact on survival. cfcDNA (**a**) or ctDNA (**b**) quantifications in copies per ml of plasma at 4 different time points during therapy. Black lines refer to median values. cfcDNA (**c**) or ctDNA (**d**) variations along 4 sampling time points during therapy. Pink triangles highlight samples with increased ctDNA level compared to the previous assessable time point. Wilcoxon tests were used to compare DNA levels. Bsl, baseline; D15, day 15; D30, day 30; Progr., disease progression. Progression free survival of patients split by ctDNA detected or not, at day 15 (**e**) and day 30 (**f**)

Matched plasma samples collected at day 15 (D15) and day 30 (D30) were available for 17 and 19 patients, respectively. At D15, all patients presented a significant decrease of both cfcDNA (Fig. 3a, *p*_bsl vs D15_ = 0.004) and ctDNA levels (Fig. 3b, *p*_bsl vs D15_ < 0.001). Reduction of cfcDNA might reflect the cytostatic effect of palbociclib on hematopoietic cells as described previously [13]. The decrease was more pronounced for ctDNA (Fig. 3c, d) and 3 patients had no more ctDNA detected at D15 (Fig. 3d). At D30, the median level of cfcDNA copies showed no significant difference compared to D15 (Fig. 3a, c). Median level of ctDNA continued to decrease between D15 and D30, with 6 patients showing undetectable rates (Fig. 3b, d). However, we observed an interpatient heterogeneity in ctDNA dynamics. More precisely, between D15 and D30, 3 patterns of ctDNA changes were observed: (i) a continuous decrease or remaining undetected ctDNA for 9 patients, (ii) a stable but detectable ctDNA level for 1 patient, and (iii) an increase of ctDNA in 5 patients (Fig. 3d, D30 pink triangles, Supplemental Table 2). Finally, at the time of radiological progression (mean time = 6 months, range = 3–24 months, at the time of analysis), all patients had detectable ctDNA levels and 79% (11/14) of them exhibited increased ctDNA levels compared to D30 (Fig. 3d, Progr. pink triangles).

Next, we assessed the impact of ctDNA detection on PFS. Detection of ctDNA at D15 (*N* = 14/17) was not associated with PFS **(***p* = 0.87, Fig. 3e). Conversely, undetectable ctDNA (*N* = 6/19) at D30 was associated with a much longer PFS than detectable ctDNA (*N* = 13/19; 25 months vs 3 months, HR = 7.2, 95% CI = 1.5–32.6, *p* = 0.004, Fig. 3f).

### ctDNA dynamics correlation with response to treatment

**Fig. 4:**
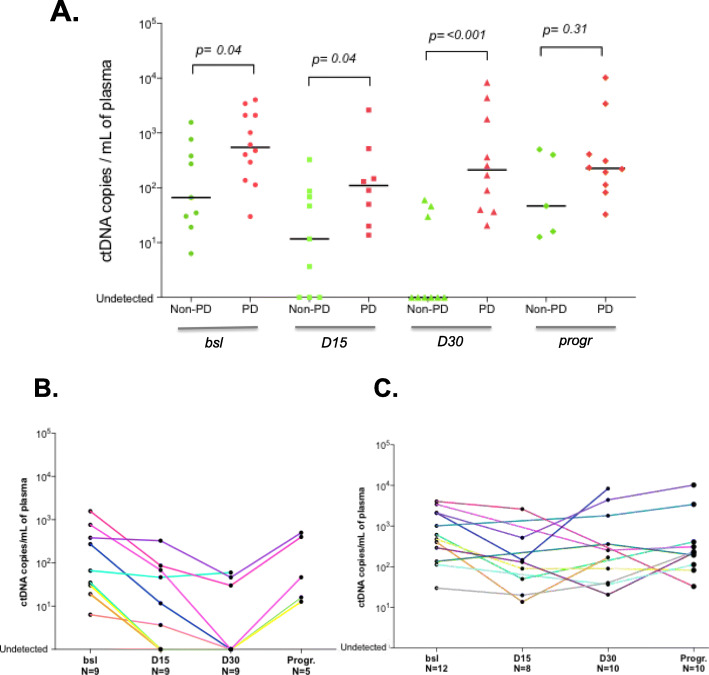
ctDNA dynamics correlation with treatment response. **a** ctDNA concentrations at the 4 sampling time points in patients with non-progressive disease (green) versus patients with progressive disease (red) after 3 months of palbociclib-fulvestrant. Black lines refer to median values. ctDNA variation in patients with non-progressive disease (**b**) or progressive disease (**c**) during treatment follow-up. Each patient is highlighted with a unique color. Bsl, baseline; D15, day 15; D30, day 30; Progr., disease progression; PD, progressive disease

We next investigated the correlation between ctDNA dynamics and response to treatment in order to estimate the potential of ctDNA quantification to monitor palbociclib-fulvestrant efficacy. To this end, we classified patients in 2 groups according to their 3-month radiological evaluation. Following the RECIST criteria, 9 patients were qualified with radiological response while 12 patients experienced disease progression (Supplemental Table 2). For the latter 12, treatment had been stopped and switched to a new line of treatment while the same treatment was maintained for the 9 patients with non-progressive disease (non-PD). First of all, we observed that the median ctDNA copy number was significantly lower in non-PD vs PD patients at baseline, D15, and D30 (Fig. 4a, Supplemental Table 2). Between D15 and D30 matched samples, we observed a rise or stabilization of ctDNA levels for 5 out of the 6 (83.3%) patients with PD (Fig. 4c, Supplemental Table 2). On the contrary, ctDNA levels remained at zero or continued to decline for 8 out of the 9 (89%) non-PD patients (Fig. 4b, Supplemental Table 2). More strikingly, at D30, all 6 patients with undetectable ctDNA had a RECIST response, whereas PD group never reached a negative ctDNA level.

We further examined the distribution of the ctDNA concentrations (copies/ml of plasma) according to the disease progression status to determine if this could be used as an early marker of treatment efficacy. We observed that the concentrations in patients with non-PD and PD overlap at all 3 time points (bsl, D15 and D30) (Fig. 4a). This underlines that ctDNA absolute value is difficult to interpret as a predictive biomarker.

### ctDNA ratios, treatment response, and impact on survival

**Fig. 5:**
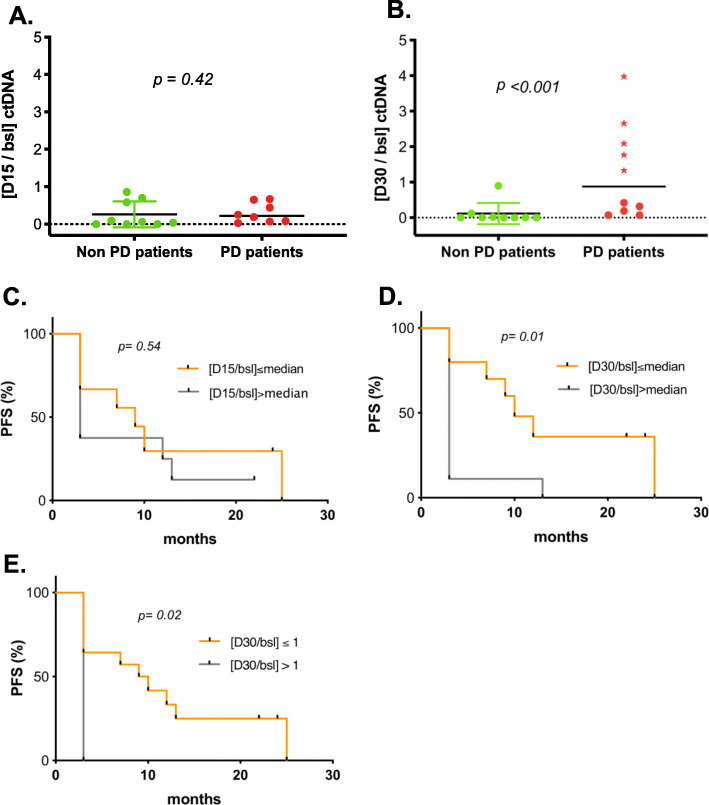
ctDNA ratios, treatment response, and impact on survival. ctDNA ratios for patients with non-progressive disease (green) vs progressive disease (red) after 15 days (**a**) or 30 days (**b**) of treatment. *P* values were calculated using Mann–Whitney tests. Median is highlighted by a solid black line. Patients highlighted with a star display increased ctDNA levels, which predict progression at 3 months. **c–e** Progression free survival of patients under palbociclib-fulvestrant therapy split by median ctDNA ratio at D15 (**c**, median D15 = 0.16), median ctDNA ratio at D30 (**d**, median D30 = 0.09), ctDNA ratio > 1 or ≤ 1 at D30 (**e**)

We therefore calculated ctDNA ratios, as previously reported by O’Leary and colleagues [13]. These ctDNA ratios correspond to the mutant allele abundance (mutant copies/ml of plasma) at a given time point relative to baseline quantification. ctDNA ratios reveal the extent of ctDNA level change during treatment. [D15/baseline] ctDNA ratios of non-PD and PD patients were all below 1, reflecting the early and systematic decline of ctDNA under palbociclib-fulvestrant treatment. At that time point (D15), the proportion of decrease shows no significant difference between the 2 groups (Fig. 5a). However, at D30, patients with non-PD always displayed decreased ctDNA levels ([D30/baseline] ctDNA ratios < 1) with a median value significantly lower than for patients with PD (median ratio D30 = 0 vs 0.9, *p* < 0.001) (Fig. 5b). It is important to note that an increase of ctDNA at day 30, highlighted by a [D30/baseline] ctDNA ratios > 1, systematically led to progression (100% *N* = 5/5) (Fig. 5b, 5 patients highlighted with a ★). This underlines the strong predictive value of the [D30/baseline] ctDNA ratio, which predicts progression at 3 months when greater than 1.

Next, we investigated whether the extent of ctDNA variation, measured by ctDNA ratios, could predict long-term outcome for patients treated with palbociclib-fulvestrant. We found that D15 ctDNA ratio was not significantly related to PFS (Fig. 5c) as opposed to what has been previously reported [13]. The dramatic reduction observed for all patients at D15 might explain the absence of significant association between [D15/baseline] ctDNA ratio and PFS in our small cohort. However, at D30, patients with ctDNA ratios above the median (*N* = 10) had shorter PFS compared the ones with ratios below median (*N* = 9) (HR = 4.1 95% CI = 1.4–12, *p* = 0.01) (Fig. 5d). Moreover, when we analyzed the PFS relative to [D30/baseline] ctDNA ratio > 1 (*N* = 5) or ≤ 1 (*N* = 14), an unbiased threshold not related to the ctDNA distribution observed in this specific cohort, the same significant impact was observed (Fig. 5e, HR = 5.1, 95% CI = 1.4–18.3, *p* = 0.02).

## Discussion

In our study, we investigated the impact of ctDNA dynamics as a predictive biomarker for palbociclib-fulvestrant efficacy in MBC. Consistently with recently published results, we found (i) no predictive effect of the baseline ctDNA level value on PFS and (ii) that all patients experienced a decline of both ctDNA and cfcDNA levels at D15. Decline of cfcDNA is specific to palbociclib, as chemotherapy treatment showed opposite trend in prior studies [7, 16–18]. This confirms the antiproliferative effect on tumor and hematopoietic cells of palbociclib.

In order to report on ctDNA dynamics and relate it to treatment response, we examined ctDNA ratio as previously defined by O’Leary et al. [13]. We did not recapitulate their association between [D15/baseline] ctDNA ratio with PFS. This might be due to the dramatic reduction observed for all patients at D15, reflecting a widespread inhibition of tumor proliferation as a consequence of treatment efficacy, as well as the small size of our cohort. However, we further analyzed D30 plasma samples for which [D30/baseline] ctDNA ratio is associated with PFS. Moreover, we demonstrated that the drop to undetectable ctDNA levels at D15 or D30 can anticipate the radiological response observed at 3 months.

Additionally, after the first decline observed at D15, all of the non-PD patients maintained a ctDNA ratio < 1 at day 30, and conversely, rising ctDNA levels at that time point predicted the radiological progression, as a consequence of the resumption of tumor proliferation. This finding goes with the fact that ctDNA levels are known to be correlated with tumor burden [7, 10].

Defining the optimum threshold to be used with a quantitative biomarker in order to discriminate positive and negative impact is essential for medical decision-making. Previous studies examining the impact of ctDNA on treatment response set up thresholds dependent on the ctDNA level distributions observed in their cohorts. O’Leary et al. used the CDR15 median, Tie et al, among their metastatic colorectal cancer cohort used ROC curves to determine the most appropriate ctDNA index and the optimal cutoff for differentiating patients with “response” and “no response,” while Garlan et al. chose specific ctDNA values as threshold in order to split their cohort in 3 prognostic classes [13, 19, 20]. However, such thresholds remain biased and difficult to apply for routine clinical practice. Our CDR15 median, for example, is quite different than the one observed by O’Leary et al. (0.09 vs 0.034). In order to increase the reproducibility, we classified our patients depending on whether they had a [D30/baseline] ctDNA ratio above or below 1, which reflects the drop or raise of ctDNA during the first month of treatment.

As an overall drop is observed at D15, our study demonstrated the importance to assess the ctDNA level at D30. This time point allowed anticipating the radiological response for 58% of patients (11/19). Indeed, patients with undetected ctDNA at that time (6/19) have shown no progression at 3 months whereas those with rising ctDNA levels (5/19) experienced radiological tumor progression. These findings might have immediate clinical impact as an earlier switch to an alternative therapy could be done after only 30 days under palbociclib-fulvestrant for about 25% of patients. However, our hypothesis-generating study requires independent validation by external studies to further confirm the clinical validity of ctDNA changes during treatment. For the remaining 8 patients (3 with non-PD and 5 with PD) whose ctDNA had decreased but remained detectable, the treatment response cannot be anticipated. Further analyses should be performed at D45, D60, or later to determine if ctDNA dynamics could discriminate them later on.

## Conclusion

We have demonstrated that early changes in ctDNA are associated with later radiological tumor responses and that serial ctDNA measurement has a significant potential to anticipate standard RECIST-based disease assessment, leading to an immediate clinical impact. Moreover, ctDNA detection and dynamics, at day 30, are prognostic factors for PFS. The conclusions drew in this exploratory study needs to be confirmed in larger cohorts. A large randomized trial, PADA-1 (NCT03079011), is currently testing the utility of real time resistant subclones detection in ctDNA from ER+ HER2− MBC treated with palbociclib and aromatase inhibitor. Serial sampling for ctDNA analysis should thus be incorporated into future clinical trials to provide a more robust assessment of this promising biomarker.

## Supplementary Information

**Additional file 1: Supplemental Figure 1.** Study workflow. **Supplemental Figure 2.** Progression free survival of patients under palbociclib and fulvestrant, split by median ctDNA value (median = 382 copies/ml of plasma) at baseline (bsl). **Supplemental Table 1.** 39 gene panel used for targeted NGS on plasma samples.

**Additional file 2: Supplementary Table 2.** Characteristics and ctDNA levels during treatment follow-up of patients carrying mutations.

## Data Availability

All the data supporting the results of this manuscript are available in Supplemental Tables 1 and 2, Supplemental Figures 1 and 2, and from the corresponding author on request.

## Abbreviations

ER+: Estrogen receptor-positive
HER2−: HER2-negative
MBC: Metastatic breast cancer
PFS: Progression-free survival
ctDNA: Circulating tumor DNA
cfcDNA: Cell-free circulating DNA
CR: Complete response
PR: Partial response
SD: Stable disease
PD: Progressive disease
Bsl: Baseline
D15: Day 15
D30: Day 30
NGS: Next generation sequencing
