# Supplementary material for: Circulating tumor DNA as a dynamic biomarker of response to palbociclib and fulvestrant in metastatic breast cancer patients
Source: Breast Cancer Res. 2021 Mar 6;23:31. doi: 10.1186/s13058-021-01411-0 (PMC7937332; doi:10.1186/s13058-021-01411-0)
Supplement: Supplementary file 1 — Additional file 1: Supplemental Figure 1. Study workflow. Supplemental Figure 2. Progression free survival of patients under palbociclib and fulvestrant, split by median ctDNA value (median = 382 copies/ml of plasma) at baseline (bsl). Supplemental Table 1. 39 gene panel used for targeted NGS on plasma samples. [file 13058_2021_1411_MOESM1_ESM.docx]

**Supplemental Figure 1.** Study workflow.

**Supplemental Figure 2.** Progression free survival of patients under palbociclib and fulvestrant, split by median ctDNA value (median = 382 copies / ml of plasma) at baseline (bsl).

| AKT1 | KRAS | STK11 |
| --- | --- | --- |
| HRAS | PIK3CA | FBXW7 |
| NOTCH2 | CTNNB1 | MTOR |
| ALK | MAP2K1 | TP53 |
| KDR | PTEN | FGFR1 |
| NOTCH4 | EGFR | NF1 |
| BRAF | MAP2K4 | TSC1 |
| KEAP1 | PTPN11 | FGFR2 |
| NRAS | ERBB2 | NFE2L2 |
| BRCA1 | MAP3K1 | TSC2 |
| KIT | RET | FGFR3 |
| PDGFRA | ERBB3 | NOTCH1 |
| BRCA2 | MET | VHL |

**Supplemental Table 1. 39 gene panel used for targeted NGS on plasma samples.**
